# Dissuasive effect, information provision, and consumer reactions to the term ‘Biotechnology’: The case of reproductive interventions in farmed fish

**DOI:** 10.1371/journal.pone.0222494

**Published:** 2019-09-26

**Authors:** Micaela M. Kulesz, Torbjörn Lundh, Dirk-Jan De Koning, Carl-Johan Lagerkvist

**Affiliations:** 1 Department of Economics, Swedish University of Agricultural Sciences, Uppsala, Sweden; 2 Department of Animal Nutrition and Management, Swedish University of Agricultural Sciences, Uppsala, Sweden; 3 Department of Animal Breeding and Genetics, Swedish University of Agricultural Sciences, Uppsala, Sweden; Universidade do Porto Instituto de Biologia Molecular e Celular, PORTUGAL

## Abstract

Biotechnology can provide innovative and efficient tools to support sustainable development of aquaculture. It is generally accepted that use of the term ‘genetically modified’ causes controversy and conflict among consumers, but little is known about how using the term ‘biotechnology’ as a salient feature on product packaging affects consumer preferences. In an online discrete choice experiment consisting of two treatments, a set of 1005 randomly chosen Swedish consumers were surveyed about use of hormone and triploidization sterilization techniques for salmonids. The information given to the treatment group included an additional sentence stating that the triploidization technique is an application of biotechnology, while the control group received the same text but without reference to biotechnology. Analysis using a hierarchical Bayes approach revealed significant consumer reactions to the term biotechnology. When the term was included in information, variation in consumer willingness-to-pay (WTP) estimates increased significantly. Moreover, some participants were dissuaded towards an option guaranteeing no biotechnological intervention in production of fish. These results have multiple implications for research and for the food industry. For research, they indicate the importance of examining the distribution of variation in WTP estimates for more complete characterization of the effects of information on consumer behavior. For the food industry, they show that associating food with biotechnology creates more variability in demand. Initiatives should be introduced to reduce the confusion associated with the term biotechnology among consumers.

## Introduction

Aquaculture (e.g., fish farming) is among the most sustainable of animal protein production systems. However, increasing competition for resources such as land and water are pushing the practice toward limits that could have negative impacts on the ecosystems[1]. Biotechnology offers applications that could help improve and optimize the aquaculture practice (involving growth, nutrition, health and reproduction), while at the same time reduce the pressure on the ecosystems. In aquaculture, most of these applications can be found on salmonid, and are typically linked to Genetic Modifications (GM).

The distinction between GM and Biotechnology is relevant for the present study. Biotechnology comprises a broad range of human interventions in biological processes in order to make those processes, or the organisms undergoing these processes, better suited to their human purpose. Genetic modification is a specific type of biotechnology where artificial changes are made to the genome of an organism to better suit its human purpose.

Salmonid–i.e. any fish belonging to the Salomonidae family, including salmons, trouts, chars, graylings and whitefishes- are indeed arguably the fish commodity group for which apparent consumption relies most on aquaculture [2]In 2016, the first biotechnology-treated salmon for final consumption was approved by the U.S. Food and Drug Administration (FDA–cf “FDA approves genetically modified fish, no label needed”, Robert Ferris, 19.11.2015).)What will be the effect of using the term biotechnology for labeling or in information directed to fish consumers?

Previous research on the effects of information provision from different sources and with different content on consumer choices has mainly focused on GM,- a subcomponent of biotechnology—and has shown that providing consumers with relevant information on GM does not necessarily increase their acceptance of GM food [3]. Furthermore, this type of information provision does not necessarily change consumer beliefs about safety concerns related to GM [4]. Instead, consumers resort to non-scientific sources to approach the conflicts entailed in the term GM [5], and make their choices based on these. Moreover, the effect of information provision on GM acceptance varies by region. For instance, Europeans are more reluctant to consume GM products than North American consumers [6].

In 2015, nearly 20 years after the first GM food product appeared in supermarkets, the U.S. FDA issued a report recognizing that the term GM continues to be questioned and accepting that it generates controversy and conflicts among consumers [7] (For a detailed discussion on consumers’ attitudes to biotechnology, the reader can refer to [8] and to [9].). Given that GM is a subcomponent of biotechnology, it can be expected that conflicts associated with the term GM are extended to the term biotechnology. However, some studies have shown that consumers can distinguish between different applications within biotechnology, provided that they receive additional information about the techniques used [10, 11, 12, 13, 14]. This suggests that consumers are open to incorporating information when linked to biotechnology, and thus that the term biotechnology is less controversial than GM.

Although previous research has been useful in identifying the importance for consumer behavior of providing consumers with additional relevant information, it gives little insight into the relative impact of the term biotechnology on its own to drive consumer decision making. Indeed, none of these studies focused on how the term ‘biotechnology’ affects consumer responsiveness to information. For example, while less controversial than GM, biotechnology may still carry different meanings for consumers, which will be reflected in their willingness-to-pay (WTP) for the product.

By facing consumers with realistic choices, Willingness to Pay (WTP) studies yield information on consumer preferences and can then be combined with information on consumers’ attitudes collected from traditional surveys. For instance,[15] compared consumers valuations of two distinct GM products across various countries, and concluded that European consumers place a higher value than the US consumers in one of the products. Recently, [16] found that consumers who are initially less opposed and knowledgeable about GM products, later on become more opposed. Since in both cases the authors conducted a WTP study, the effects of the differences could be measured, and consumers’ preferences could be assessed.

This study examined how using the term biotechnology in product information affects consumer WTP for salmonids. Specifically, it examined the consequences of including the term biotechnology as part of the information on salmonid product packaging for the heterogeneity of consumer WTP distribution.

## Material and methods

### Study background

Farmed fish are raised in cages, from where they may escape into the wild. The escaped fish eventually breed with wild fish and create a hybrid fish that can contaminate the wild gene pool or even outcompete native strains. To date, hormone treatment has provided a non-biotechnological technique to prevent genetic contamination from occurring, by sterilizing the fish. Hormonal sex reversal is the most widely used biotechnological method in the production of tropical fishes. The resulting fish are all of the same sex and, while they are not necessarily infertile, this limits the risk of genetic contamination of local stocks with escapes. However, there are concerns about hormone residues in the environment with widespread use of this treatment.

A recently developed, but not yet commercially available, application of biotechnology in aquaculture relates to preventing farmed and wild fish from crossbreeding. In triploidization, the eggs of the fish are pressure-treated just after fertilization. This prevents that the second polar body is squeezed out from the egg (so-called extrusion, which normally completes the second meiotic division) and thus results in a triploid embryo with 3n chromosome number instead of a diploid with 2n chromosome number. The embryo then develops as if it had not been treated, but with three sets of chromosomes. Female triploids are completely infertile, as they do not mature sexually. Triploid males develop gonads as normal, but are generally considered to be fully infertile. As no foreign DNA is introduced into the embryo and no changes are made to the DNA itself, triploid fish are not considered GM and do not require special labeling under current regulations of the European Parliament and of the Council (EC—Regulation (EC) No 1829/2003 of the European Parliament and of the Council of 22 September 2003 on genetically modified food and feed.). Furthermore, there are additional benefits for aquaculture from adopting triploids, as managing mono-sex populations is easier and can be more profitable if one of the sexes grows faster and/or more efficiently. Compared with hormone treatment, the triploidization technique has direct and indirect benefits for the consumer, since it carries no risk of residual hormones, and for the environment, since it offers better protection against genetic contamination of wild stocks.

### Study design

Data were collected using an online survey consisting of two parts: (1) a questionnaire and (2) a choice experiment. A representative sample of 1005 Swedish consumers was selected from a Swedish consumer panel provided by the marketing company GfK NORM. Given the scope of the research, a set of screening questions excluded those consuming frozen or fresh fish less than twice a month and those working in fish-related industries, such as fisheries research institutions, private aquaculture firms, etc.

After pilot testing with 10 participants, the survey was conducted during October 2016. Each participant received an e-mail with a link to the experiment. Respondents received remuneration from the marketing company according to normal standards.

### Sample descriptives

The first part of the questionnaire comprised socio-demographic questions relating to age, gender, occupation, income level, area of residence, and household size. Table 1 shows the characteristics of the sample and the average for the population of Sweden (A copy of the questionnaire can also be found on S2 File.).

**Table 1:** Socio-demographic characteristics of the study sample and of the Swedish population as a whole.

Variable	Description	Sample	Swedish population
**Gender**	Male (base)	43.18%	49.80%
	Female	56.82%	50.20%
**Age**	Average (in years)	47	41
**Income**	Average household income (in SEK)	30 001–40 000	25 000–28 999
**Area**	Place of residence		
**LargeCity**	City (>150 000 inhabitants)	35.62%	37.13%
**MedCity**	Urban/medium-sized city (50 000–150 000 inhabitants)	29.25%	34.56%
**RuralDvt**	Rural development (<50 000 inhabitants)	35.12%	28.31%
**Household size**	Average number of individuals per household	2.35	2.60

In general, the demographical variables were close to the Swedish average (cf European Social Survey, Round 8).

The gender distribution was slightly skewed towards females, the income distribution slightly larger, while differences in age, area of residence, and household size differences were minor.

#### Attitudes

The second part of the questionnaire asked respondents about: (1) general attitudes (using a binary measure) to the impact of science on food quality and the environment, because such attitudes can be expected to influence the cognitive processing of information related to biotechnology innovations; and (2) interest and perceptions related to fish consumption.

Respondents’ interest and perceptions about fish were evaluated using a 7-point Likert scale (1 = Strongly disagree to 7 = Strongly agree). Following [17] scores 5, 6, and 7 were collapsed into “Yes”, and all other scores were collapsed into “No”. Table 2 shows the detailed questions and the results.

**Table 2:** Attitudes to science and interest in fish consumption among study participants.

Variable	Description	Yes	No
**a) ScienceFood**	Science has mostly had a positive impact on the quality of food	56.82%	43.18%
**b) InterestFish**	I have a strong interest in fish consumption	47.36%	52.64%
**c) FarmedFishSafe**	Farmed fish is safe to consume.	37.91%	62.09%

#### Discrete choice experiment

A between-sample discrete choice experiment (CE) was used, because the triploidization technique is not yet in commercial use. In the CE, respondents were asked to choose among two different choices for fish that differed with regard to three attributes, namely (1) origin; (2) sterilization technique used (hormones/triploidization); and (3) price (all prices are expressed in Swedish crowns (SEK). In October 2016, 100 SEK were equivalent to 10.40 Euros). Table 3 describes the attributes and the levels. The number of attributes was kept to a minimum in relation to the purpose of the study [18]. This is relevant to the reliability of the welfare estimates, since [19] found that the stability of preferences for cue attributes is affected by the number of attributes. Thus we defined the attributes to capture consumer reactions to the term biotechnology. The attribute ‘origin’ was included to complete the set that all possible fish consumers could expect to experience, including non-farmed fish (i.e., wild fish harvested from the oceans (or lakes) and not subjected to any type of treatment). Farmed fish can be treated with hormones or triploidization, or simply reared in cages. In Sweden, only “None” is currently sold. The base price level (130 SEK/kg) was defined based on current market prices for farmed fish, and the remaining two price levels represented linear transformations of the base price. The price range was set so as to span the price range for farmed and wild salmonids in Sweden at the time of the study.

**Table 3:** Attributes and levels.

Attribute	Levels	Description
**Origin**	Farm	Fish raised in cages in the ocean or in the sea
	Wild	Naturally-born fish harvested in the wild
**Sterilization Technique**	None	Farmed fish not subjected to any sterilization technique
	Hormones	Farmed fish sterilized using hormone treatment
	Triploid	Farmed fish sterilized using the triploidization technique
**Price**	130 SEK/kg	
	260 SEK/kg	
	390 SEK/kg	

The choice experiment was preceded by a background text (see S1 File) describing the background information as specified in subsection 2.1. The purpose of the text was two-fold: (1) It provided context to explain the different sterilization techniques to the respondents; and (2) it formed the basis for our two treatments. Based on previous findings on attitudes to biotechnology [8] and the impact on consumption (Section 1), we defined the following two treatments, and randomly assigned participants to each:

#### Control

The background text described the triploidization technique without characterizing it as biotechnology.

#### Treatment

The background text described the triploidization technique and contained one extra sentence explaining that triploidization is a technique from biotechnology. The content was expressed in a value-neutral tone.

**Fig 1:**
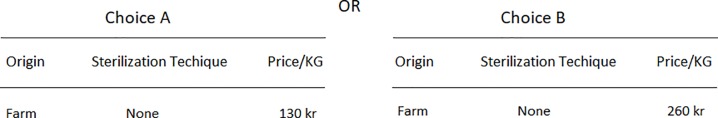
Example of a choice situation in the choice experiment.

Using a full factorial design, participants were faced with 66 choice situations. The design was restricted because the origin ‘Wild’ could only occur in combination with the sterilization technique ‘None’. Therefore, only the origin ‘Farm’ was combined with the three different levels of the attribute sterilization technique. Both levels for the origin attribute were combined with the three price levels. As “Wild” cannot be combined with Triploid or Hormones, we are left with 12 (2*3*3–2*3) choices. These are paired to all the alternatives except the own, which gives a total of 66 choices (12*11/2). Fig 1 illustrates a choice situation.

You are asked to make a decision between two options. Please indicate whether you prefer to buy Choice A or Choice B.

The number of choice alternatives faced by the respondents may raise concerns regarding fatigue, as suggested by [20] and [21]. However, a study by [22] revisited the issue of respondent fatigue in repeated choice settings and found that the amount of difference in error variance from larger numbers of choice sets was often small and had little influence on substantive model results. Moreover, [23] showed that there is little loss of reliability and validity from using a larger number of choice tasks. In fact, the literature suggests that considerable gains can be achieved by increasing the number of choice tasks per respondent, such as generation of learning effects which increase model structure reliability and precision [24, 25, 26]. It has been reported that a similar increase in model precision can be obtained by increasing the number of tasks as by proportionally increasing the number of respondents [26]. That said, we randomized the order of the choices within respondents “[to break] the correlation between scale variation across tasks and attributes of the tasks” ([22]:page 628).

Following [27], we did not include an opt-out alternative in the choice experiment. The main reason for this goes to the study purpose of examining trade-offs between fish production techniques, rather than estimating market shares. Furthermore, due to the screening questions used, respondents could be expected to have to face this type of choice when buying fish. Hence, the use of an opt-out alternative would be misleading.

## Model specification and estimation procedures

### Model specification

Random utility theory (RUT) provides a family of probabilistic choice models which describe how choice probabilities relate to changes in choice tasks (i.e., attributes and their levels) and to individual choosers [28, 29]. In modeling the nested data structure of respondents who completed a set of choice tasks, with each task including two choice concepts, the general structure of the mixed logit model used was defined according to [30] as:

$$U_{int}=\beta \;X_{int}+\eta_{int}+\epsilon_{nit}$$ (1)

where individual *n* faces a number of choice situations, *t*, where s/he has to make a choice between two or more alternatives, *i*, where *η*_*int*_ varies randomly over individuals and *ε*_*nit*_ varies randomly over alternatives, and thus its variance cannot be identified separately from *β*. The flexibility of this model is related to the underlying distributions: (1) *η*_*int*_ can assume any general distribution, such as normal, lognormal, etc. (*θ*, *κ*); and (2) *ε*_*nit*_ is *i*.*i*.*d*. extreme value (Gumbel). The choice probability in a mixed logit model is then:

$$P_{int}=\int \frac{e^{\left(X_{nit}\beta +\eta_{int}\right)}}{\sum_{i=1}^{I}e^{\left(X_{nit}\beta +\eta_{int}\right)}}\;f(\frac{\eta }{\Theta })d\eta$$ (2)

To explain the source of heterogeneity in preferences and choices, we extended our model by including individual characteristics (*D*_*i*_) and attitudes variables (*A*_*i*_) in our model specification. Following [17], our specification was augmented with interactions:

$$U_{int}=X_{int}\beta +\psi (X_{int}*D_{i})+\zeta (X_{int}*A_{i})\eta_{int}+\epsilon_{nit}$$ (3)

Therefore, the choice probability function to be estimated became:

$$P_{int}=\int \frac{e^{\left(X_{int}\beta +\psi (X_{int}*D_{i})+\zeta (X_{int}*A_{i})\right)}}{\sum_{i=1}^{I}e^{\left(X_{int}\beta +\psi (X_{int}*D_{i})+\zeta (X_{int}*A_{i})\right)}}\;f(\frac{\eta }{\Theta })d\eta$$ (4)

As explained in [31], *f* can take the form:

$$f_{1}(\beta_{1}){(1,f}_{2}(\beta_{2}),\ldots ,f_{k}(\beta_{k}))$$ (5)

When this transformation of *f* was used for estimations, it was understood that the estimations were taking place in the ‘WTP space’. If not, they were taking place in the ‘preference space’.

### Data analysis and estimation procedures

We implemented a Hierarchical Bayes (HB) approach [32, 33, 34] for computing Eq 4 in WTP space(estimations were computed in STATA 14 using the command Bayesmixedlogitwtp developed by [35]). The HB approach uses Markov Chain Monte Carlo techniques to specify the posteriors. It estimates a distribution of parameters and uses information at individual choice level to calculate individual specific parameters (or posterior estimates). A number of studies have already used HB to estimate discrete choice models (e.g., [30, 36, 37, 38, 39, 40]. To perform these calculations, HB uses an iterative process that converges after a certain number to draws from the posterior distribution (Draws for the sample are obtained using Gibbs sampling, and draws for the individual coefficients are taken from Metropolis-Hastings algorithm). Iterations before convergence are known as ‘burns’ and are not considered in the calculation. Furthermore, after convergence, only every *g*^*th*^ iteration is used for the calculations.

As [33] and [36] point out, due to the Bernstein-von Mises theorem, the posterior Bayesian distributions asymptotically converges to a normal distribution. This is the same as the asymptotic distribution of the maximum-likelihood estimator used in the classical estimation. However, for the present study, estimating individual marginal utility parameters using Bayesian statistics has the main advantage of significantly reducing computation times.

Current research disagrees on the differences between a Bayesian and a classical approach, but the findings are not conclusive when it comes to using these approaches in the preference space or in the WTP space [37, 41]. Estimating in the WTP space directly potentially yields more stable estimates [31], particularly when the price parameter is strictly positive. Because the latter was the case in our specification, we implemented our estimations in the WTP space directly.

We used the data from the survey and the CE to study consumer WTP for triploid fish as follows: (1) We used a log-normal distribution for the negative-price coefficients and a normal distribution for the other parameters; (2) since we did not have strong reason to assume that individuals’ responses differ depending on their socio-economic background, we assumed all parameters to be random [42]; (3) priors were taken to be non-informative, i.e., the Bayesian procedures start with values set to zero; and (4) we employed 20 000 iterations, but only used that last 10 000 to start in equilibrium (or closest to it), and only every 10th observation was kept after convergence [43].

In addition to the attribute variables, other factors may affect the decision process. A natural extension of the model to be estimated was thus to consider respondents’ demographic characteristics and their perceptions and attitudes (see sections 2.2.1. and 2.2.2.).

We report the estimated means of the coefficients in Table 4 and the standard deviations in S1 Table. The attribute levels that served as the basis are as follows: (1) Origin: Farmed; (2) Price: Lowest; and (3) Sterilization Technique: None.

For the reader that would like to expand on the technical aspects of this section, we refer to [41, 44, 45].

**Table 4:** Willingness-to-pay (WTP) estimates: Mean coefficients in 0.01SEK.

	Control (no information provided on Triploid as biotechnology)		Treatment (information provided on Triploid as biotechnology)	
	WTP	Std. Err.	WTP	Std. Err.
**Price**	-3.353***	(0.274)	-3.718***	(0.317)
**Wild**	-0.176***	(0.038)	0.048	(0.064)
**Hormones**	-0.183***	(0.041)	0.031	(0.064)
**Triploid**	-0.162***	(0.032)	-0.092	(0.061)
***Interaction with demographic variables***				
Female*Wild	0.016	(0.031)	0.159**	(0.063)
Age*Wild	0.038	(0.020)	0.046	(0.039)
Income*Wild	0.057	(0.040)	0.005	(0.059)
LargeCity*Wild	0.047	(0.039)	-0.341***	(0.071)
Female*Hormones	0.070**	(0.028)	0.031	(0.064)
Age*Hormones	-0.002	(0.015)	0.007	(0.032)
Income*Hormones	0.048	(0.030)	0.099	(0.066)
LargeCity*Hormones	0.073**	(0.037)	0.023	(0.063)
Female*Triploid	0.015	(0.034)	-0.040	(0.075)
Age*Triploid	0.019	(0.015)	0.067	(0.041)
Income*Triploid	-0.004	(0.034)	0.056	(0.075)
LargeCity*Triploid	0.048	(0.031)	-0.141**	(0.066)
***Interaction with attitudes variables***				
ScienceFood*Wild	-0.097***	(0.036)	0.016	(0.077)
InterestFish*Wild	0.135***	(0.035)	0.273***	(0.068)
FarmedFishSafe*Wild	-0.349***	(0.034)	-0.221***	(0.060)
ScienceFood*Hormones	0.065**	(0.032)	-0.027	(0.068)
InterestFish*Hormones	0.150***	(0.038)	0.172**	(0.067)
FarmedFishSafe*Hormones	-0.115***	(0.033)	-0.037	(0.062)
ScienceFood*Triploid	0.032	(0.032)	-0.096	(0.062)
InterestFish*Triploid	0.245***	(0.042)	0.236***	(0.062)
FarmedFishSafe*Triploid	-0.211***	(0.036)	-0.188**	(0.080)
**Number of Individuals**	685		320	
**Number of Observations**	90420		42238	
**Draws/Burns**	20000/10000		20000/10000	

*** to 1%

** to 5%

## Results

### Mean estimates by information treatment

Table 4 shows the mean WTP estimates for the control and the treatment groups. For both samples we found that the coefficients for price were negative, which indicates that on average respondents prefer less expensive fish.

#### Control

The coefficient for wild fish was negative, indicating that consumers who were not informed about the use of biotechnology evaluated farmed fish positively, and were not willing to pay for wild fish if the price difference with farmed fish was greater than ~18 SEK/kg. The coefficients corresponding to the sterilization techniques (hormones and triploid) were also negative, indicating that on average consumers prefer to consume fish that has not been treated. In terms of magnitudes, consumers were not willing to pay for a fish treated with hormones if the price difference with the fish that has not been treated was greater than ~18.5 SEK/kg. The magnitude is of ~16 SEK/kg for the difference between triploid fish and fish that has not been treated.

The demographic background of consumers was irrelevant to the assessment of preferences for the origin and for the sterilization technique (Table 4). Indeed, only place of residence and gender were significant for assessing fish treated with hormones (We would here like to point out that interaction effects need to be read together with the main effects. So, for instance, the coefficient for female consumers consuming Hormones result from adding together the coefficient for Female*Hormones together with the coefficient for Hormones, which is overall negative.). In both cases the final effect was negative, suggesting that female consumers and consumers living in large cities are less likely to consume fish treated with hormones. Last, the attitudinal variables were of relevance to explain consumers’ preferences. Specifically, respondents with a strong interest in fish consumption were willing to pay a premium for wild fish and for fish treated with hormones or triploid. Additionally, consumers who perceived the impact of science on food to be positive were more likely to consume farmed (and non-treated) fish than wild fish, which corroborates findings by [46] and [47]. Finally, consumers who believed that farmed fish was safe to consume were less likely to choose wild, hormones, or triploid fish.

#### Treatment effects

In the treatment sample, we observed no mean differences in consumer preferences for wild or for treated fish. Regarding consumers’ demographic background, we observed that female consumers were more prone to pay a premium for wild fish, an observation also suggested in the control group. Moreover, people living in larger cities appeared to be more likely to buy farmed fish. Consumers living in large cities were, in general, willing to pay a premium for non-treated farmed fish. These results corroborate previous findings suggesting a heuristic of familiarity in food consumption [48], whereby people living in large cities would need to go to special stores to find wild fish and thus most of the fish they consume is non-treated farmed fish available at most ordinary retail stores. Additionally, those consumers with strong interest in fish consumption were willing to buy treated and wild fish and pay a premium for it. Moreover, and consistently with their preferences, consumers who believed that farmed fish is safe to consume preferred to buy non-treated farmed fish. The main difference with the control related to those consumers who believe that science has a positive impact on the quality of the food. In the treatment we observed non-significant coefficients, suggesting that the presence of the term biotechnology may be controversial even for those consumers biased towards the positive impact of science.

### Effect of information treatment on WTP distribution

#### Consumers’ relative preferences for triploid and wild

**Fig 2:**
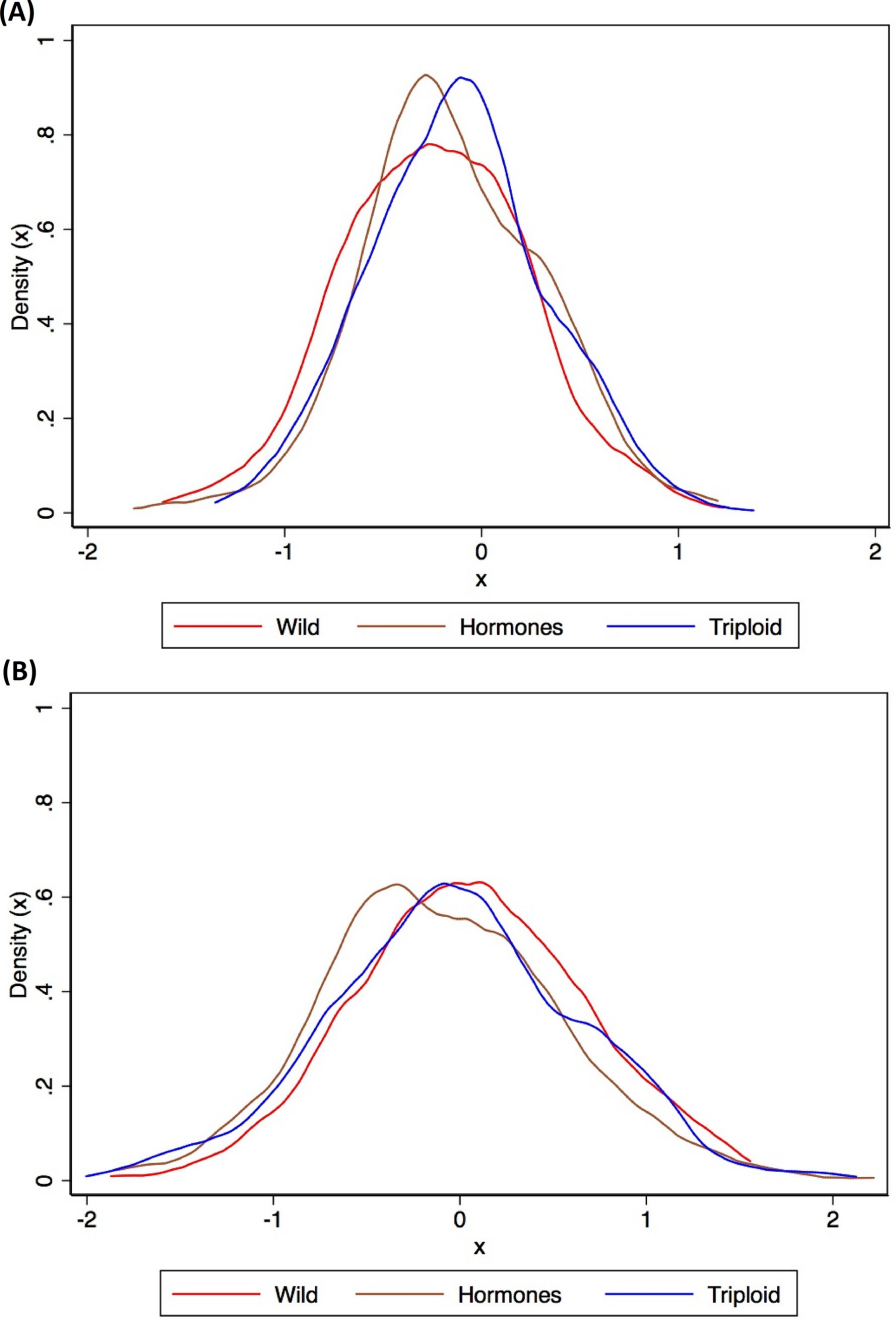
Density distribution of individual WTP coefficients for wild, hormones and triploid. (a) In the control group. (b) In the treatment group.

Fig 2 shows the density distribution of the mean coefficients for origin Wild, and sterilization techniques Hormones and Triploid corresponding to the control and the treatment, respectively.

- Control
- Treatment

The shape of the distributions in Fig 2 captures how unobserved heterogeneities affect consumer choices. After mean-normalizing the distributions, the Kolmogorov-Smirnov (k-s) test showed that: a) the distributions for Triploid and Wild were significantly different from each other for both samples (Control: *combined k-s*: 2.0033, *p* = 0.000; Treatment: *combined k-s*: 2.6653, *p* = 0.000); b) the distributions for Hormones and Wild were significantly different from each other (Control: *combined k-s*: 2.0902, *p* = 0.000; Treatment: *combined k-s*: 2.6817, *p* = 0.000); and c) the distributions for Hormones and Triploid were significantly different from each other (Control: *combined k-s*: 2.0902, *p* = 0.000; Treatment: *combined k-s*: 2.6117, *p* = 0.000). These results suggest that (1) adding the term biotechnology to describe Triploid does not have an impact on consumers’ relative evaluations of the products and that (2) unobserved heterogeneity affects the final price consumers are willing to pay for Wild, Hormones, and Triploid fish.

### Characterizing triploid as biotechnology increases the heterogeneity in WTP

**Fig 3:**
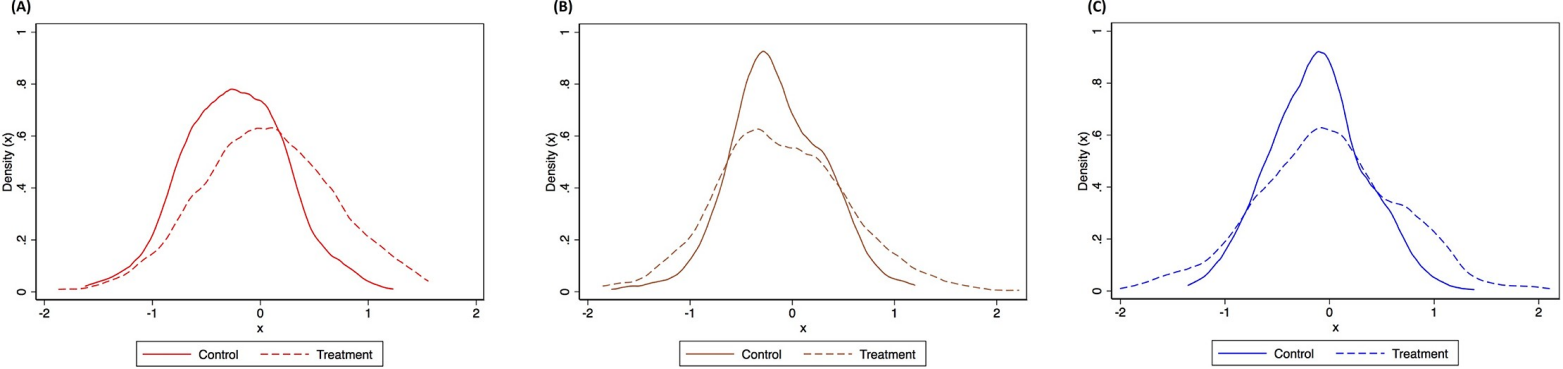
Density distribution of individual WTP coefficients (x) for the control and treatment groups. (a) For Wild. (b) For Hormones. (c) For Triploid.

The effect of clarifying that the Triploid technique is biotechnology increased the overall unobserved heterogeneity in our estimates (S1 Table). To further illustrate this finding, in Fig 3 we plot the distributions of the mean WTP coefficients for the control and the treatment, by origin and sterilization technique. For both Wild and Triploid, the mean differences between WTP in the control and in the treatment were significant (Wild—*t*: -8.3621, *p* = 0.000; Triploid—*t*: -2.7700, *p* = 0.0029), with lower WTP in the control. However, for Hormones the differences were not significant (Hormones—*t*: -1.2526, *p* = 0.1053), suggesting that the treatment mainly affected consumer WTP for Wild and Triploid fish. Additionally, the respective standard deviations exhibited significant differences among samples for the three cases (Wild—*f*: 0.6139, *p* = 0.000; Hormones—*f*: 0.5176, *p* = 0.000; Triploid—*f*: 0.4625, *p* = 0.000) and were greater for the treatment group. Moreover, for the treatment, the mean WTP for Wild fish was significantly higher (by on average around 65%) than for both Hormones and Triploid fish. This suggests that linking biotechnology to Triploid also affected consumer preferences for the other techniques, since consumers shifted towards wild fish, but the WTP for hormones remained rather unaffected by linking Triploid to biotechnology.

- Wild
- Hormones
- Triploid

Furthermore, from Fig 3 it can be seen that the variation in the estimates increased for the treatment group (on average by nearly 18%). These results suggest that introducing the term biotechnology in relation to triploidization: (i) generates a larger variance in consumer demand for all products; and (ii) generates a substitution effect (i.e., demand for wild fish increases). Given that the term biotechnology was the only difference between the control and the treatment samples, it can be assumed that treatment differences were related to the presence of this word, and thus that consumers react to the meaning they assign to the use of this term. Fig 3 suggests that, in the treatment, some consumers were dissuaded towards wild fish instead of treated farmed fish. The mere presence of the term ‘biotechnology’ linked to one type of farmed fish only was thus enough to deter some consumers from choosing treated farmed fish.

## Discussion

The main contribution of the present study is in describing the effect of information provision on consumer preferences. The study was based on a design that captured consumer WTP for wild salmonids and for farmed salmonids sterilized using two alternative techniques, based on an information treatment making the term ‘biotechnology’ salient for one type of sterilization.

The findings indicate that (1) in general, consumers prefer a less expensive and non-treated product, which corroborates existing research related to GM-treatment of other food products [49, 50]; (2) linking the sterilization technique to the term biotechnology affects mean WTP and significantly increases the variation in consumer WTP estimates; and (3) introduction of the term biotechnology in relation to aquaculture affects the demand for both farmed and wild fish, suggesting noticeable reactions to the term biotechnology. This corroborates the expectation voiced by public authorities in the US when use of biotechnology was approved for use in aquaculture [7].

In our design, the information treatment induced a potential conflict in relation to use of biotechnology in production of farmed fish. On the one hand, use of triploidization as the biotechnology could provide an alternative to hormone treatment (and thus has potential to be perceived as beneficial for food-related health). On the other hand, there could be concerns about the biotechnology in itself. This is apparent on comparing the mean individual WTP in the treatment sample: the average individual WTP for the wild fish was significantly higher (by around 65%) than for both types of treated fish. However, the results suggest that the mere presence of this one term induced a propensity among respondents to move away from treated farmed fish and choose fish from the wild instead. This change-of-preference result indicates that a general positive image about wild fish might be reinforced when consumers face an unknown product, which is in line with findings by [51]. Previous research on products from aquaculture have reported that consumer perception favors wild over farmed fish in terms of taste, healthiness, and nutritional value [52, 53], even though it is generally accepted that fish cannot be differentiated in terms of the latter two [54]. Following [53], this gap between perceptions and facts can be explained by lack of knowledge among consumers about aquaculture and by their opinions being driven by affect rather than rationale.

The finding that part of the effect of providing information about biotechnology relates to the variance and shape of the WTP distributions is relevant for further research related to consumer preference formation on novel product features and for the aquaculture industry. The results from this study suggest that providing results in terms of mean WTP and the like is insufficient. Instead, in order to understand preference formation, attention should be given to the distribution of WTP for product attributes and levels. The information entailed in these distributions provides a more complete understanding of preference formation. That said, individual WTPs will depend on whether subjects associate the term biotechnology with a positive meaning or with a non-positive meaning (in line with [55]). Our results indicated that individuals with prior and positive opinions about biotechnology were most likely less affected by the presence of the term, and thus more prone to choose Triploid fish, whereas individuals with no prior and negative opinions about biotechnology were most influenced by the presence of the term and did not choose Triploid fish. The final effect of including biotechnology, which we arbitrarily call ‘*dissuasive effect*’, will thus depend on the relative size of the two segments of respondents, and the strength of the positive effect of one segment compared with the non-positive effect of the other segment [56]. All of these can be observed by studying the distribution of variation in WTP estimates.

Our findings provide valuable insights for the aquaculture industry. Increasing the supply of farmed fish from aquaculture is currently the only alternative to wild fish and the current world production of fish must be maintained without altering the product’s nutritional and health qualities. Consequently, should consumers wish to maintain their fish consumption levels, they will most likely have to shift their demand towards farmed fish. Within recent decades, more sustainable and potentially less invasive methods for growing farmed fish have emerged with the use of biotechnology, namely triploidization. The product is expected to reach the market eventually, and assessing consumers’ preferences for this new product is a promising step.

In Sweden, current labeling options for fish indicate whether it was produced in a sustainable manner and state whether it comes from the Baltic Sea. Eventually, when it becomes apparent that aquaculture is driving the supply of fish, consumers will demand additional information on the method of production before making their purchasing decisions. When this occurs, the results from this study suggest that, on average, wild fish will be favored, at a premium. However, on studying the distribution of individual unobserved heterogeneities we found support for yet another claim: providing consumers with additional information that involves inclusion of a controversial term, such as biotechnology, can enhance the positive image of the conventional product (e.g., wild fish) to the extent that a ‘dissuasive effect’ is triggered, and consumers are dissuaded away from the new product (e.g., triploid fish).

## Conclusions

This study indicates that using the term biotechnology for labeling or in information directed to fish consumers can have a dissuasive effect on consumption and this can be attributed to the different meanings consumers assign to the term. For some respondents, the presence of this one term is enough to shift their preferences to an option free of interventions (here wild fish). This is the one option for which every consumer shares the same understanding since, objectively, it is the product in its most natural form. For other respondents, e.g., those who believe that science has a positive impact on food quality, fish from biotechnology is not disregarded as an option. Scientific knowledge is known to be positively correlated with perceptions on biotechnology, which indicates that when consumers base their choices on scientific rationality, concerns about the perceived unnaturalness of the product lose importance.

Based on the variation in WTP distributions, this study showed that the mere presence of the term biotechnology in the example used affected consumer behavior by enhancing the positive image of wild fish, not affecting the average WTP for fish treated with hormones, and dissuading a segment of consumers away from fish sterilized by triploidization. The final effect of the dissuasive effect will ultimately depend on the variation in WTP for wild, hormones and triploid fish by different segments of consumers. From the results obtained in the present study, it can be concluded that adding the term biotechnology as a salient feature on the packaging of salmonids will generate more variation in demand. Thus it should be combined with efforts to reduce confusion about the term biotechnology among consumers.

## Supporting information

S1 File Background text. (DOCX)

S2 File Questionnaire. (DOCX)

S1 Table (DOCX)
