# Supplementary material for: Dissuasive effect, information provision, and consumer reactions to the term ‘Biotechnology’: The case of reproductive interventions in farmed fish
Source: PLoS One. 2019 Sep 26;14(9):e0222494. doi: 10.1371/journal.pone.0222494 (PMC6762195; doi:10.1371/journal.pone.0222494)
Supplement: S1 File — (DOCX) [file pone.0222494.s001.docx]

**S1 - Appendix A**

**Background Text (Original text in Swedish)**

The extra sentence is added in red fonts for information purposes only.


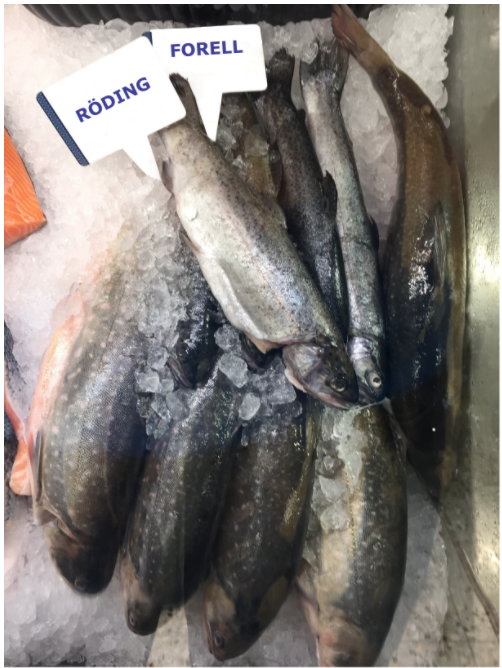
Vattenbruket är den del av matproduktionen som växer snabbast globalt sett enligt FN:s matorgan FAO. För första gången konsumeras nu mer protein från vattenbruksprodukter än från boskap i världen. Allt eftersom världens befolkning fortsätter att växa de kommande årtiondena och den globala levnadsstandarden höjs, kommer efterfrågan på fisk att fortsätta öka. Eftersom det mesta av fisket av vilda fiskar redan är helt exploaterat, måste mycket av den nya efterfrågan mötas från vattenbruket. Odlad fisk är resurseffektiv proteinkälla och nyttig mat. Sverige har mycket goda förutsättningar för en hållbar matfiskproduktion.

Produktionen av regnbåge och röding sker i dagsläget nästan uteslutande i odlingar med öppna kassar. Ska röding och regnbåge kunna finnas tillgänglig både för restaurang och konsument måste den stora efterfrågan mötas med produktionsökning av odlad fisk. Vid produktion av odlad fisk finns alltid en potentiell risk för att fisk rymmer eller av annat skäl kan ta sig ut från odlingsplatsen. Det kan påverka den vilda fisken negativt eftersom det finns risk att den odlade fisken kan reproducera sig med vild fisk av samma art. För att möjliggöra fiskodling måste fiskodlaren säkerställa att det inte kan förekomma eller kraftigt minska risken för reproduktion mellan odlad och vild fisk.

Det finns 2 alternativ för att lösa detta problem, vilka båda skapar fiskar som saknar reproduktionsförmåga. De två sätten är (1) **Hormonbehandling** gör att all fisk i odlingen får samma kön, och (2) **Triploidbehandlin**g. Respektive metod förklaras nedan:

(1) **Hormonbehandlingen** innebär att fisken före tre veckors ålder behandlas med ett hormon så att alla fiskar blir av kvinnligt kön. Behandlingen sker genom att småfisken får simma i vattentankar med en utblandad hormonlösning. Att alla fiskar får kvinnligt kön minskar men utesluter inte risken att odlad fisk reproducerar sig med vild.

(2) **Triploidbehandling** är modern bioteknik inom livsmedelskedjan går ut på att rom från fisken behandlas med värme och tryck. Denna behandling ger 3 könskromosomer hos varje fisk (istället för de två kromosomer som normalt finns). På detta sätt blir fiskarna sterila och kan då inte reproducera.

Det finns inga kända negativa miljöeffekter av denna behandling eftersom:

- (a) De odlade fiskarna saknar reproduktionsförmåga.
- (b) Fisken inte behandlas med någon substans som kan spridas till eller påverka den omgivande miljön.

I denna undersökning som vi ber dig delta i finns 4 typer av fisk att välja mellan:

- Odlad röding och/eller regnbåge som inte har behandlats för att minska risken för att den ska kunna reproducera sig med vild röding.
- Odlad röding och/eller regnbåge som är hormonbehandlad.
- Odlad röding och/eller regnbåge som har triploidbehandlats.
- Fångad vild röding och/eller regnbåge (som inte är behandlad) men som kan vara en korsning mellan odlad och vild fisk.

Det är inte möjligt att känna någon skillnad i smak beroende på hur fisken har behandlats för att minska risken för förökning med vilt-levande fisk. Inte heller fiskens utseende påverkas av vilken behandlingsmetod som används. Smakskillnader mellan odlad och vilt fångad laxfisk kan förekomma beroende på att odlad fisk föds upp på ett speciellt foder.

**Background Text (content translation in English for review purposes only)**

The extra sentence is here added in red fonts for information purposes only.

Industrial fish farming is used to deliver a specific product to consumers and to help protect wild fish populations from overfishing. In Sweden, farmed fish (mainly salmonids, such as arctic char and rainbow trout) are raised in pens, from where they can escape into the wild. The escaped fish eventually breed with wild fish and create a hybrid that can bring pure wild fish to extinction: the more farmed fish escape and breed with wild fish, the less wild fish will be left.

To reduce the impacts of the escaped farmed fish on the wild population, industries prevent them from breeding through: (1) hormonal sex reversal (*Hormones*) or (2) triploidization (*Triploid*).

1. *Hormones* involves adding high doses of hormones to the water when fish are 3 weeks of age, so that they reverse their sex to male. However, after sex reversal they can still breed with wild female fish. Moreover, this procedure is very environmentally unfriendly, since the hormones are spread in the water and from there to the rest of the environment (including those organisms that eat fish).
2. *Triploid* is an application of modern biotechnology in the food production chain. It involves temperature and pressure treatment of fish eggs just after fertilization. The fish then grow normally, but with three sex chromosomes, which completely prevents reproduction: the escaped fishes cannot breed with wild fish. This technique does not have negative effects on the environment, since the fish are not treated with any solution that can cause damage to the environment (including consumers).

In all, consumers have the choice to buy any of the following:

1. Farm fish not exposed to any sterilization technique and fed pelleted food.
2. Farm fish exposed to hormonal sex reversal and fed pelleted food.
3. Farm fish sterilized through triploidization and fed pelleted food.
4. Wild fish not exposed to any sterilization technique, but could be hybrid fish.

Consumers cannot taste the difference between wild and farm fish, regardless of the treatment received (if any).
