# Supplementary material for: Dissuasive effect, information provision, and consumer reactions to the term ‘Biotechnology’: The case of reproductive interventions in farmed fish
Source: PLoS One. 2019 Sep 26;14(9):e0222494. doi: 10.1371/journal.pone.0222494 (PMC6762195; doi:10.1371/journal.pone.0222494)
Supplement: S2 File — (DOCX) [file pone.0222494.s002.docx]

**S2 – Appendix B**

**Questionnaire (Original language and English)**

1. Är du?

Are you?

| Man  Man |
| --- |
| Kvinna  Woman |

1. Hur gammal är du?

How old are you?

1. Ungefär hur stor är hushållets sammanlagda månadsinkomst före skatt?

What is (approximately) your household's total monthly income before tax?

| <= 10 000 kr |
| --- |
| 10 001–20 000 kr |
| 20 001–30 000 kr |
| 30 001–40 000 kr |
| 40 001–50 000 kr |
| 50 001–60 000 kr |
| 60 001–70 000 kr |
| >= 70 000 kr |

1. Vilket av nedanstående stämmer bäst överens med var du bor?

Your place of residence is…?

| ROW: |
| --- |
| Storstad (ort med fler än 150 000 invånare)  Large (>150 000 inhabitants) |
| Tätort/medelstor stad (50 000–150 000 invånare)  Urban/medium-sized city (50 000-150 000 inhabitants) |
| Glesbygd (färre än 50 000 invånare)  Rural development (<50 000 inhabitants) |

1. Hur många personer bor i ditt hushåll, inklusive dig själv?
   How many people live in your household, including yourself?
