# Supplementary material for: Dissuasive effect, information provision, and consumer reactions to the term ‘Biotechnology’: The case of reproductive interventions in farmed fish
Source: PLoS One. 2019 Sep 26;14(9):e0222494. doi: 10.1371/journal.pone.0222494 (PMC6762195; doi:10.1371/journal.pone.0222494)
Supplement: S1 Table — (DOCX) [file pone.0222494.s003.docx]

**S1 - Table**

**Table 5: Willingness-to-pay (WTP) estimates: Standard deviations**

|  | **Control**  (**no information provided**  **on Triploid as biotechnology**) | | **Treatment**  **(information provided on Triploid as biotechnology)** | |
| --- | --- | --- | --- | --- |
|  | WTP | Std. Err. | WTP | Std. Err. |
| **Price** | 0.397 | (0.064) | 0.507 | (0.099) |
| **Wild** | 0.198 | (0.032) | 0.361 | (0.071) |
| **Hormones** | 0.192 | (0.032) | 0.375 | (0.082) |
| **Triploid** | 0.191 | (0.034) | 0.374 | (0.073) |
| ***Interaction with demographic variables*** |  |  |  |  |
| **Female*Wild** | 0.202 | (0.036) | 0.379 | (0.073) |
| **Age*Wild** | 0.160 | (0.040) | 0.295 | (0.078) |
| **Income*Wild** | 0.200 | (0.039) | 0.376 | (0.073) |
| **LargeCity*Wild** | 0.193 | (0.030) | 0.391 | (0.072) |
| **Female*Hormones** | 0.193 | (0.030) | 0.401 | (0.073) |
| **Age*Hormones** | 0.099 | (0.018) | 0.207 | (0.044) |
| **Income*Hormones** | 0.198 | (0.034) | 0.383 | (0.083) |
| **LargeCity*Hormones** | 0.195 | (0.028) | 0.391 | (0.070) |
| **Female*Triploid** | 0.198 | (0.033) | 0.386 | (0.071) |
| **Age*Triploid** | 0.114 | (0.024) | 0.230 | (0.050) |
| **Income*Triploid** | 0.192 | (0.032) | 0.372 | (0.075) |
| **LargeCity*Triploid** | 0.199 | (0.035) | 0.376 | (0.072) |
| ***Interaction with attitudes variables*** |  |  |  |  |
| **ScienceFood*Wild** | 0.194 | (0.032) | 0.387 | (0.080) |
| **InterestFish*Wild** | 0.197 | (0.034) | 0.388 | (0.074) |
| **FarmedFishSafe*Wild** | 0.207 | (0.035) | 0.380 | (0.071) |
| **ScienceFood*Hormones** | 0.196 | (0.033) | 0.373 | (0.071) |
| **InterestFish*Hormones** | 0.197 | (0.034) | 0.392 | (0.076) |
| **FarmedFishSafe*Hormones** | 0.195 | (0.033) | 0.385 | (0.076) |
| **ScienceFood*Triploid** | 0.194 | (0.033) | 0.387 | (0.078) |
| **InterestFish*Triploid** | 0.203 | (0.037) | 0.373 | (0.071) |
| **FarmedFishSafe*Triploid** | 0.197 | (0.034) | 0.371 | (0.068) |
| **Number of Individuals** | 685 | | 320 | |
| **Number of Observations** | 90420 | | 42238 | |
| **Draws/Burns** | 20000/10000 | | 20000/10000 | |
